# The Leptomeninges Produce Prostaglandin D_2_ Involved in Sleep Regulation in Mice

**DOI:** 10.3389/fncel.2018.00357

**Published:** 2018-10-11

**Authors:** Yoan Cherasse, Kosuke Aritake, Yo Oishi, Mahesh K. Kaushik, Mustafa Korkutata, Yoshihiro Urade

**Affiliations:** ^1^International Institute for Integrative Sleep Medicine (WPI-IIIS), University of Tsukuba, Tsukuba, Japan; ^2^Ph.D. Program in Human Biology, School of Integrative and Global Majors, University of Tsukuba, Tsukuba, Japan; ^3^The University of Tokyo Hospital, The University of Tokyo, Tokyo, Japan

**Keywords:** prostaglandin D_2_, leptomeninges, lipocalin-type prostaglandin D synthase, sleep, adeno-associated virus (AAV)

## Abstract

Injection of nanomolar amounts of prostaglandin D_2_ (PGD_2_) into the rat brain has dose and time-dependent somnogenic effects, and the PGD_2_-induced sleep is indistinguishable from physiologic sleep. Sleep-inducing PGD_2_ is produced in the brain by lipocalin-type PGD_2_ synthase (LPGDS). Three potential intracranial sources of LPGDS have been identified: oligodendrocytes, choroid plexus, and leptomeninges. We aimed at the identification of the site of synthesis of somnogenic PGD_2_ and therefore, generated a transgenic mouse line with the LPGDS gene amenable to conditional deletion using Cre recombinase (flox-LPGDS mouse). To identify the cell type responsible for producing somnogenic PGD_2_, we engineered animals lacking LPGDS expression specifically in oligodendrocytes (OD-LPGDS KO), choroid plexus (CP-LPGDS KO), or leptomeninges (LM-LPGDS KO). We measured prostaglandins and LPGDS concentrations together with PGD synthase activity in the brain of these mice. While the LPGDS amount and PGD synthase activity were drastically reduced in the OD- and LM-LPGDS KO mice, they were unchanged in the CP-LPGDS KO mice compared with control animals. We then recorded electroencephalograms, electromyograms, and locomotor activity to measure sleep in 10-week-old mice with specific knockdown of LPGDS in each of the three targets. Using selenium tetrachloride, a specific PGDS inhibitor, we demonstrated that sleep is inhibited in OD-LPGDS and CP-LPGDS KO mice, but not in the LM-LPGDS KO mice. We concluded that somnogenic PGD_2_ is produced primarily by the leptomeninges, and not by oligodendrocytes or choroid plexus.

## Introduction

In 1982, it was discovered that microinjecting prostaglandin (PG) D_2_ into the preoptic area of conscious rats induces sleep (Ueno et al., 1982). It is now widely accepted that PGD_2_ (Urade and Hayaishi, 2010; Huang et al., 2011) is one of the endogenous chemicals proposed by Ishimori (Ishimori, 1909; Kubota, 1989) and Pieron (Legendre and Pieron, 1913) more than 100 years ago to induce sleep. Other substances that induce sleep are cytokines (Krueger et al., 2011), adenosine (Porkka-Heiskanen et al., 1997; McCarley, 2007; Huang et al., 2011; Porkka-Heiskanen and Kalinchuk, 2011), anandamide (Garcia-Garcia et al., 2009), and peptides such as urotensin II (Huitron-Resendiz et al., 2005). These studies independently postulated that prolonged periods of wakefulness can lead to an accumulation of hypothetical endogenous substances that induce sleep. Indeed, elevated PGD_2_ levels are found in diseases with sleep alterations such as mastocytosis and African trypanosomiasis (Roberts et al., 1980; Pentreath et al., 1990).

Prostaglandin D_2_ is a derivative of arachidonic acid produced by two different PGDS, hematopoietic PGDS, and LPGDS. Hematopoietic PGDS is a member of the sigma class glutathione-S-transferase family (Urade et al., 1987) and synthesizes PGD_2_, for example, in mast cells during allergic reactions (Tanaka et al., 2000), whereas glutathione-independent LPGDS is primarily expressed in the brain (Urade and Hayaishi, 2000). Natural sleep is inhibited in wild-type and hematopoietic PGDS knockout (KO) mice, but not LPGDS KO mice, after administering the inorganic tetravalent selenium compound selenium tetrachloride (SeCl_4_), a specific inhibitor of PGDS activity (Islam et al., 1991; Matsumura et al., 1991; Takahata et al., 1993), demonstrating that PGD_2_ is involved in regulating physiologic sleep (Qu et al., 2006). A recent study from our lab showed that postictal (pathological) sleep is also regulated via LPGDS-derived PGD_2_ (Kaushik et al., 2014).

Experiments based on *in situ* hybridization and immunohistochemistry in rat brains demonstrated that LPGDS is expressed in three intracranial cell populations: cells of the CP, cells of the LM, and OD (Urade et al., 1993). Which of these cell population produces the PGD_2_ involved in sleep-wake regulation is, however, unclear.

In the present study, we generated a mouse line with a loxP-site-inserted LPGDS gene that is amenable to conditional disruption by cell type-specific expression of Cre recombinase to obtain CP-LPGDS KO mice, LM-LPGDS KO mice, and OD-LPGDS KO mice. When the mice were administered the PGDS inhibitor SeCl_4_, only the CP- and OD-LPGDS KO mice exhibited disrupted sleep, and not the LM-LPGDS KO mice. Our findings reveal that the LM, but not the CP and OD, produce the PGD_2_ that induces physiologic sleep.

## Materials and Methods

### Genetic Mouse Models

Animals were handled according to the NIH Guide for the Care and Use of Laboratory Animals and in accordance with protocols approved by animal research committees at the Osaka Bioscience Institute and the International Institute for Integrative Sleep Medicine (animal protocol #16-086). All male mice (weighing 24–28 g, 10–14 weeks old) used in the present study were housed at a constant temperature (22 ± 1°C) with a relative humidity of 50 ± 2% on an automatically controlled 12:12 light/dark cycle (light on at 8:00 am). A mouse line called flox-LPGDS on a C57BL/6 background with a loxP-site-inserted LPGDS gene that is conditionally disrupted by expressing Cre recombinase was generated as previously described (Kaneko et al., 2012) and used in this study. This mouse line has not been deposited to any animal repository.

### Generation of Cell Type-Specific LPGDS Knockout Animals

Several serotypes of AAV have been identified and are commonly used in neuroscience. These serotypes differ in their tropism (the types of cells they infect), making AAV a very useful system for preferentially targeting the gene of interest in specific cell types. We tested the ability for AAV to specifically infect the LM and the CP. We tested 5 different serotypes of AAVs (serotypes 2, 5, 8, 10, and 11) expressing the reporter protein mCHERRY in wild-type mice and discovered that the AAV of serotype 5 was the most efficient to target the CP, while only the serotype 8, when injected in postnatal mice (2-day-old), could infect the LM. Therefore LM-LPGDS KO mice were obtained by micro-injecting 6 μL of serotype 8 AAV-Cre into the lateral ventricle of 2-day-old flox-LPGDS mice. Briefly, neonatal mice were anesthetized by hypothermia on ice for 5 min before fixing to the pad of a stereotaxic arm. A glass micropipette with a 10- to 20-μm-diameter tip was introduced manually into the external corner of the right eye of the animal. Light pressure was applied to pass through the eye socket bone and deliver the AAV vectors into the lateral ventricle (Kalamarides et al., 2002). Using an air pressure injection system, 6 μL of viral vector from serotype 8 was delivered into the CSF over 3 min. After the injection, the pipette was kept in place for a few seconds until the CSF pressure returned to a normal level and then removed. Following the injection, the neonatal mice were kept in a cage warmed to 37°C until they recovered and then returned to their mother.

CP-LPGDS KO mice were obtained by micro-injecting serotype 5 AAV-Cre into the lateral ventricle of adult male flox-LPGDS mice. Briefly, mice weighing 24–28 g were anesthetized with pentobarbital (50 mg/kg, i.p.), and 50 μl AAV5-Cre was stereotaxically microinjected into the left lateral ventricle (0.45 mm caudal to bregma, 1.6 mm lateral from bregma, and 1.6 mm below the dural surface) at a flow rate of 0.6 μL/min using a 100-μL Hamilton syringe and a syringe pump. Our observations indicate that the virus can disperse across ventricles and infect the remaining CP.

OD-LPGDS KO mice were obtained by cross-breeding flox-LPGDS females with transgenic mice expressing Cre recombinase under control of the nestin promoter. The nestin promoter drives Cre expression only in neural precursor cells, leading to total KO of LPGDS in the derived cells, including the OD, but not in the LM or CP.

As controls, in the case of LM- and CP-LPGDS KO mice we used littermate Flox-LPGDS mice injected with AAVs of the same serotypes and administered following the same protocol, however, these AAVs express the fluorescent protein mCHERRY instead of Cre recombinase (hence called littermate control mice). In the case of OD-LPGDS KO mice, littermate control mice are born from the same parents but did not express Cre under the control of Nestin promoter (*n* = 6 for each group).

### Vigilance State Assessment Using Electroencephalogram (EEG), Electromyogram (EMG) and Locomotor Activity Recordings

Vigilance states were assessed in adult male conditional LPGDS KO mice as described earlier (Huang et al., 2001). All mice subjected to EEG recordings were injected with vehicle or drug on two consecutive days. On day 1, the mice were injected with vehicle (saline, i.p.) at 10 am, and the 24-h recordings performed on day 1 were used as baseline data. On day 2, the mice were injected with SeCl_4_ (i.p., 5 mg/kg, 10 mL/kg body weight) and EEG/EMG signals were recorded for 24 h. The EEG/EMG signals were amplified and filtered (EEG: 0.5–30 Hz, EMG: 20–200 Hz), then digitized at a sampling rate of 128 Hz, and recorded using SLEEPSIGN software (Kohtoh et al., 2008). In addition, locomotor activity was recorded with an infrared photocell sensor (Biotex, Kyoto, Japan). The vigilance state of each 10-s epoch was automatically scored offline into three stages: waking, REM sleep, and non-REM sleep, according to standard criteria (Mizoguchi et al., 2001). As a final step, defined vigilance stages were examined visually, and corrected manually when necessary. A total of 6 to 8 animals per group were used in this experiment.

### Generation of AAV Vectors

AAV-CMV-Cre and AAV-CMV-mCherry (mCHE) vectors from serotypes 5 and 8 were obtained as described earlier (Lazarus et al., 2011). Briefly, the AAV-mCHE vector plasmid was generated by replacing the humanized *Renilla reniformis* green fluorescent protein (hrGFP) sequence from the AAV-hrGFP vector plasmid with the mCHE sequence. Subsequently, the gene coding for mCHE was replaced with the Cre recombinase coding sequence derived by polymerase chain reaction from the pBS185 plasmid (Sauer and Henderson, 1990). The serotype 5 AAV was generated by tripartite transfection (AAV-pXR5 capsid plasmid, adenovirus helper plasmid, and AAV-vector plasmid), as well as the serotype 8 AAV (AAV-rep2/cap8 capsid plasmid, adenovirus helper plasmid, and AAV-vector plasmid) into HEK293-derived AAV-293 cells (Stratagene, catalog #240073). Three days after transfection, the virus was extracted and then quantified by quantitative polymerase chain reaction.

### Immunohistochemistry

Following all procedures, the animals were deeply anesthetized with chloral hydrate (500 mg/kg, i.p.) and perfused through the left ventricle of the heart with saline followed by neutral buffered 10% formalin. The brains were removed and placed in 20% sucrose in phosphate-buffered saline (PBS) overnight at 4°C for cryoprotection. The brains were then frozen on dry ice and sectioned at 30 μm on a freezing microtome. Immunohistochemistry was performed on free-floating sections with a primary antibody directed at LPGDS as described previously (Beuckmann et al., 2000). Briefly, sections were rinsed in PBS, incubated in 3% hydrogen peroxide in PBS for 30 min at room temperature, and then sequentially at room temperature in 3% normal donkey serum and 0.25% Triton X-100 in PBS (PBT) for 1 h and primary antibody diluted in PBT with 0.02% sodium azide overnight. After several washes in PBS, the sections were incubated in goat anti-rabbit horseradish peroxidase-conjugated secondary antibody for 1 h, and the presence of LPGDS was revealed by a 0.05% 3,3′-diaminobenzidine/0.015% hydrogen peroxidase reaction for 10 min. Photomicrographs were obtained using a Nikon Eclipse E600 microscope coupled to a Digital Sight DS-2Mv camera. LM, CP, and OD were characterized by their localization in the tissue sections, as well as their morphology and their potential expression for LPGDS.

### Enzymatic PGDS Assay

The effect of the conditional KO of LPGDS (*n* = 6 for each group) in LM, CP, and OD on the brain PGDS enzymatic activity was measured by incubating 40 μg of brain extracts at 25°C for 1 min with [1-^14^C]PG H_2_ (final concentration of 40 μM) in 50 μL of 0.1 M Tris–HCl (pH 8.0) containing 1 mM dithiothreitol (Urade et al., 1995). [1-^14^C]PG H_2_ was prepared from [1-^14^C]arachidonic acid (2.20 GBq/mmol; PerkinElmer, Wellesley, MA, United States) as described previously (Urade et al., 1985).

### Prostaglandin D_2_, Enzyme Immunoassays

The brains of LM-LPGDS KO, CP-LPGDS KO, and OD-LPGDS KO mice (*n* = 6 for each group) were harvested and immediately frozen in liquid nitrogen. They were then homogenized in ethanol containing 0.02% HCl at pH 2.0 and centrifuged at 500 g for 20 min. ^3^H-Labeled PGD_2_ (60 Bq/assay; PerkinElmer) was added to the supernatant as tracer to estimate recovery. The recovery value was approximately 60%. PGD_2_ was extracted with ethyl acetate, which was evaporated under nitrogen. The samples were then separated by HPLC (Gilson, Middleton, WI, United States) (Pinzar et al., 2000). PGD_2_ was quantified using a PGD_2_ enzyme immunoassay kit for this prostanoid (Cayman Chemicals, Ann Arbor, MI, United States).

### Statistical Analysis

Data are presented as the mean ± standard error of the mean (SEM). For the sleep data analysis, an unpaired Student’s *t*-test was used to analyze the amount of time spent in the different sleep-wake states. A one- or two-way ANOVA followed by the Fisher protected least significant difference test was used to determine whether differences in LPGDS, PGD_2_ contents, or PGDS activity in the brain of control or KO mice were statistically significant. In all cases, *p* < 0.05 was considered statistically significant.

## Results

### Generation of Cell Type-Specific LPGDS KO Mice

**FIGURE 1:**
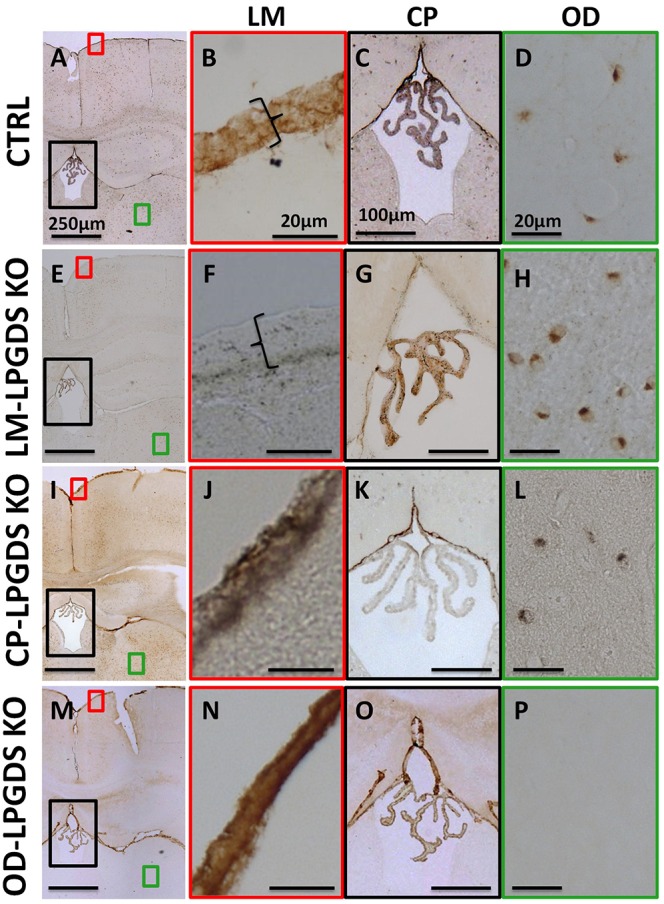
Localization of LPGDS expression in the LPGDS^flox^ control (CTRL) mice **(A)**, LM-LPGDS KO mice **(E)**, CP-LPGDS KO mice **(I)**, and OD-LPGDS KO mice **(M)** by immunohistochemistry. Serial coronal sections were stained with an LPGDS antibody and representative images are presented. LPGDS was not detected in the LM of LM-LPGDS KO mice **(F)** while it was strongly expressed in the LM of LPGDS^flox^ control mice **(B)** and other cell type-specific KO mice **(J,N)**. LPGDS staining was absent from CP cells in CP-LPGDS KO mice **(K)**, but was expressed in the other mice **(C,G,O)**. No LPGDS staining was detected in the brain parenchyma of the OD-LPGDS KO mice **(P)** where many OD, morphologically identified, were positive for LPGDS staining in the other mice **(D,H,L)**.

To determine which intracranial cell population produces the PGD_2_ that regulates sleep, we generated cell type-specific LPGDS KO mice for the CP, OD, and LM based on a mouse line in which the LPGDS gene is flanked by loxP sites in intron 1 and 6 and is amenable to conditional deletion by Cre recombinase (**Supplementary Figure S1**) (Kaneko et al., 2012). To obtain the LM-LPGDS KO mice, we injected 6 μL of AAV serotype 8 expressing Cre recombinase (AAV8-Cre) into the CSF of 2-day-old mice. Two months later, immunohistochemistry confirmed the absence of LPGDS expression in the LM, but not in the CP or OD (**Figures 1E–H**). To generate CP-LPGDS KO mice, we infused 50 μL of AAV-Cre, serotype 5 (AAV5-Cre), into the lateral ventricle of adult animals and 3 weeks later immunostaining confirmed the selective absence of LPGDS in the CP (**Figures 1I–L**). Moreover, we crossed our floxed LPGDS mice with mice expressing Cre under control of the rat Nestin promoter and enhancer (Tronche et al., 1999). The Nestin promoter drives Cre recombinase expression only in cells from the brain parenchyma of neuroectodermal origin (neurons, astrocyte, and OD), and among them only the OD express LPGDS (Urade and Hayaishi, 2011). This resulted to the deletion of LPGDS in the OD (OD-LPGDS KO mice; **Figures 1M–P**). In contrast, LPGDS immunostaining in the control flox-LPGDS animals revealed intense staining for LPGDS in the LM, CP, and OD (**Figures 1A–D**).

### LPGDS and PGD_2_ Content in the Brain of LM-, CP-, and OD-LPGDS KO Mice

**FIGURE 2:**
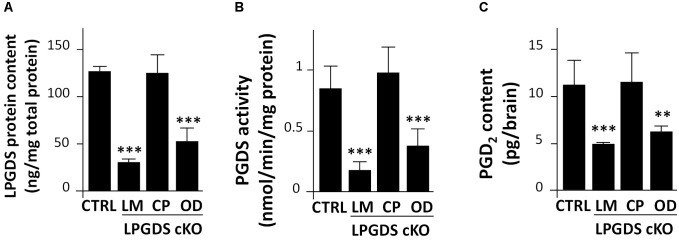
Functional analysis of LPGDS disruption in the three conditional LPGDS KO mice. **(A)** Relative LPGDS protein content in the brain of LPGDS^flox^ (CTRL), LM-LPGDS KO, CP-LPGDS KO, and OD-LPGDS KO mice. **(B)** PGDS activity in the brain of CTRL and cell type-specific KO mice. **(C)** PGD_2_ content in the brain of CTRL and cell type-specific KO mice. Values are means ± SEM (*n* = 6). ^∗∗^*P* < 0.01; ^∗∗∗^*P* < 0.001 compared with LPGDS^flox^ (CTRL), one-way ANOVA followed by the Fisher protected least significant difference test.

First, we measured LPGDS concentration in the brain of the LM-, CP-, and OD-LPGDS KO mice. We then determined the amount of PGD_2_ in flox-LPGDS mice compared with that in control mice. Whereas the total amount of LPGDS in the brain of the CP-LPGDS KO mice remained unchanged compared with the control mice (**Figure 2A**), the amount of LPGDS was reduced by 58.3 ± 11.1% (*p* < 0.001) and 75.7 ± 2.7% (*p* < 0.001), respectively, in the OD- and LM-LPGDS KO mice compared with the control mice. These results indicate that the contribution of the CP to the production of LPGDS in the mouse brain was negligible. In contrast, one-third to one-half of the brain LPGDS was produced by the OD.

We next measured PGDS enzymatic activity of the LM-, CP-, OD-LPGDS KO mice and their respective control littermates. PGDS activity in the brain of the CP-LPGDS KO mice remained unchanged compared with the control animals (*p* = 0.6082), whereas it was significantly reduced by 39.7 ± 6.5% (*p* = 0.0028) and 78.9 ± 6.9% (*p* = 0.0004), respectively, in the OD- and LM-LPGDS KO mice (**Figure 2B**). These findings are in good agreement with the distribution of LPGDS in the brain of the LM-, CP-, and OD-LPGDS KO mice.

Finally, we measured the total amount of PGD_2_ contained in the brain of the cell type-specific KO animals. In the OD- and LM-LPGDS KO mice, PGD_2_ concentrations were significantly reduced by 44.2 ± 4.9% (*p* = 0.004) and 55.5 ± 1.8% (*p* = 0.001), respectively, compared with the control flox-LPGDS animals (**Figure 2C**). On the other hand, no change in PGD_2_ concentration was observed in the brain of CP-LPGDS KO mice compared with the control mice (*p* = 0.9078).

### SeCl_4_-Induced Insomnia Was Abolished in LM-, but Not in OD- or CP-LPGDS KO Mice

**FIGURE 3:**
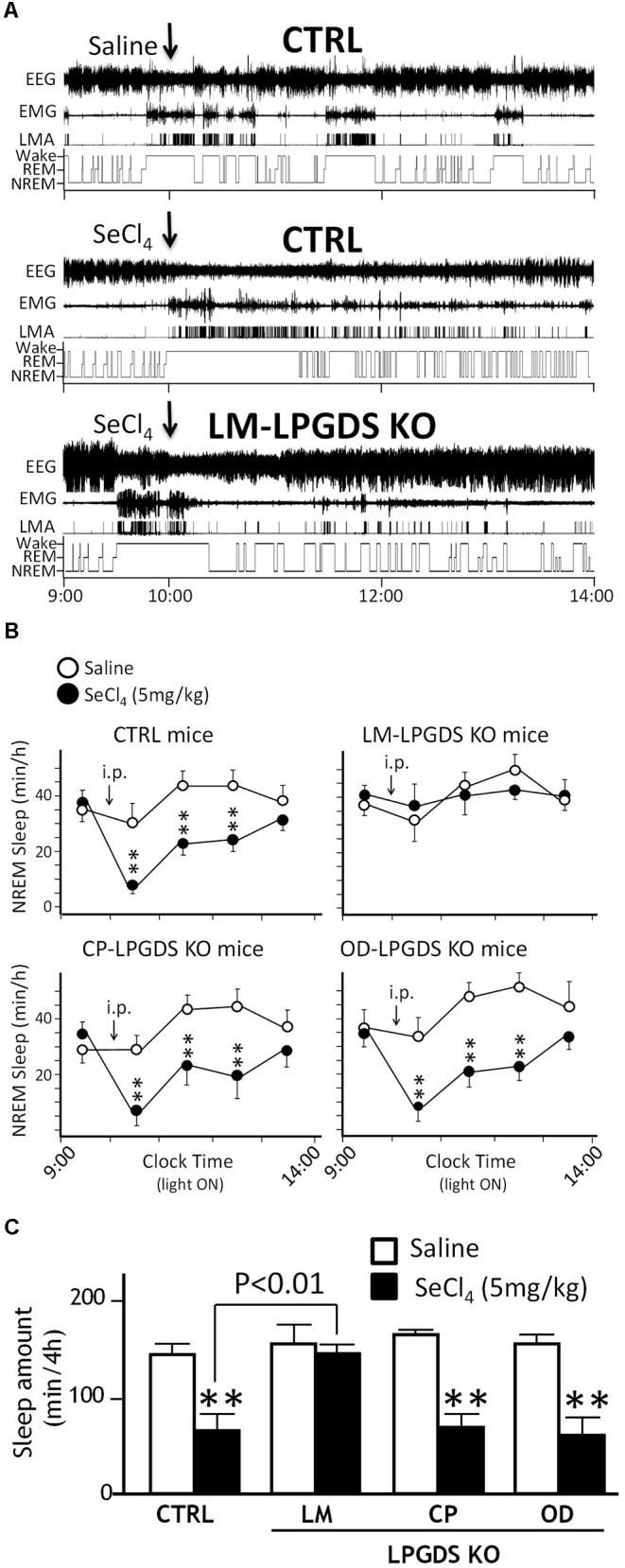
SeCl_4_ inhibited sleep in LPGDS^flox^ control mice (CTRL), CP-LPGDS KO mice, and OD-LPGDS KO mice, but not in LM-LPGDS KO mice. **(A)** Typical examples of EEG, EMG, and locomotor activity (LMA), and hypnograms after administration of saline (vehicle) or SeCl_4_ in LPGDS^flox^ control and LM-LPGDS KO mice. **(B)** Injection of SeCl_4_ significantly *(decreased the hourly amount of sleep during the subsequent 3 h compared with saline in LPGDS^flox^ control mice, CP-LPGDS KO mice, and OD-LPGDS KO mice, but not in LM-LPGDS KO mice. **(C)** Injection of SeCl_4_ dramatically decreased the total amount of sleep during the subsequent 4 h compared with saline in LPGDS^flox^ control mice, CP-LPGDS KO mice, and OD-LPGDS KO mice, but not in LM-LPGDS KO mice. Values are means ± SEM (*n* = 6–8). ^∗∗^*P* < 0.01 compared with its own control (saline).)*

We analyzed the sleep-wake pattern of the LM-, CP-, and OD-LPGDS KO mice by measuring their EEG and EMG activity before and after an i.p. injection of the PGDS inhibitor SeCl_4_ at a dose of 5 mg/kg at 10:00 am when mice are mostly asleep, as previously described. SeCl_4_ is a specific inhibitor of PGDS activity that can inhibit natural sleep in wild-type and hematopoietic PGDS KO mice, but not LPGDS KO mice (Qu et al., 2006). The basal sleep behavior of the cell type-specific LM-, CP-, and OD-LPGDS KO mice was identical to that of their control littermates, except for the LM-LPGDS KO mice, which exhibited moderately reduced REM sleep activity during the daytime (-20.4 ± 5.8%, *p* = 0.0487; **Supplementary Figure S2**). Furthermore, we analyzed EEG/EMG recordings obtained during a 4-h period after i.p. injections of SeCl_4_ (5 mg/kg) and vehicle. The total amount of time spent in sleep was reduced in the CP- and OD-LPGDS KO mice as well as in the control mice during the 3-h period following the injection of SeCl_4_ compared with vehicle, but in the LM-LPGDS KO mice, the time spent in sleep after the injection of SeCl_4_ was indistinguishable from that following the vehicle injection (**Figure 3A** and **Supplementary Figure S3**).

The changes in the time-course of sleep in the cell type-specific CP-, OD-, and LM-LPGDS KO mice during the 4 h following the i.p. injection of 5 mg/kg SeCl_4_ is shown in **Figure 3B**. The total amount of sleep was drastically reduced in the CP- and OD-LPGDS KO mice during the 4 h following the SeCl_4_ injection (-50.1 ± 11.6%, *p* = 0.004 and -52.7 ± 8.9%, *p* = 0.0022, respectively) compared with the vehicle control (**Figure 3C**). The sleep amount was similarly reduced by the injection of SeCl_4_ (-59.3 ± 3.1%, *p* < 0.0001) in the control animals (flox-LPGDS). By contrast, no sleep reduction was observed in the LM-LPGDS KO mice, indicating their complete insensitivity to the SeCl_4_ injection (-2.5 ± 9.4%, *p* = 0.7341). These results suggest that LPGDS expressed in the LM, but not the CP or OD, is involved in the regulation of physiologic sleep.

## Discussion

The administration of an LPGDS inhibitor (SeCl_4_) or PGD_2_ receptor, subtype DP_1_, antagonist (ONO-4127Na) inhibits sleep in rats and mice, indicating that the PGD_2_ system is crucial for maintaining physiologic sleep (Qu et al., 2006). The specific intracranial cell population expressing LPGDS and producing the PGD_2_ responsible for the induction of sleep, however, has been debated over the last 30 years (Narumiya et al., 1982; Hayaishi, 1991; Urade et al., 1993; Vesin et al., 1995; Yamashima et al., 1997; Qu et al., 2006; Urade and Hayaishi, 2011; Zhang et al., 2017a,b). Using cell type-specific LPGDS KO mice for the LM, OD, or CP, we here identified the LM as the primary source of LPGDS involved in the regulation of sleep. Therefore, LPGDS in the LM produces PGD_2_, which is secreted into the CSF, where it stimulates DP_1_ receptors as a sleep hormone. In the same pathway, adenosine is then released as a secondary sleep-promoting messenger and activates adenosine A_2A_ receptor-expressing neurons (Lazarus et al., 2012; Zhang et al., 2017a). Through this pathway, the sleep center in the ventrolateral preoptic nucleus is subsequently activated and the histaminergic arousal center in the tuberomammillary nucleus is reciprocally regulated by the primary sleep-promoting neurons in the ventrolateral preoptic nucleus via GABAergic inhibitory projections (Sherin et al., 1998; Chung et al., 2017). Where DP_1_ and A_2A_ receptors are expressed, however, remains to be clarified.

The basal sleep behavior of the cell type-specific KO mice for the CP and OD was identical to that of their control littermates, while the LM-LPGDS KO mice exhibited slightly reduced REM sleep activity during the daytime. The absence of a major sleep phenotype in the conditional LPGDS KO mice is in good agreement with previous reports of global LPGDS KO mice (Hayaishi et al., 2004). The most widely accepted explanation so far involves a compensatory mechanism during embryonic development that helps the animal to recover a normal sleep pattern, as sleep is essential for the survival of animals. In the LM-PGDS KO mice, disruption of LPGDS occurs in a late-stage of development, which may explain the incomplete compensation and the moderate REM sleep abnormality.

While using AAVs expressing Cre recombinase permitted to delete the expression of LPGDS in the CP and the LM, we cannot exclude the possibility that other cell types might also have been infected and their copy of *lpgds* gene deleted. Indeed, other cells such as tanycytes -a special ependymal cells found in the third ventricle of the brain and on the floor of the fourth ventricle- or ependymocytes -a type of glial cell forming the ependyma, a thin neuroepithelial lining the ventricular system- were most likely in contact with the AAVs and might have been infected. However, it is unclear if the serotypes of AAVs used in this study can effectively infect such ependymal cells. Furthermore, even if these and other cell types have also been infected, then started to express Cre recombinase, and were deleted of their *lpgds* gene, we believe that the effect would have remained unnoticed. To our knowledge, these cells have never been reported to express LPGDS and therefore animals should not be impacted by the potential ectopic expression of Cre recombinase.

## Conclusion

Lipocalin-type PGDS is implicated in the production of PGD_2_ that is involved in the regulation of physiologic sleep. Our findings in cell type-specific LPGDS KO mice demonstrate that the somnogenic PGD_2_ is primarily produced by LPGDS in the LM.

## Data Availability Statement

The raw data supporting the conclusions of this manuscript will be made available by the authors, without undue reservation, to any qualified researcher.

## Author Contributions

YC and YU designed the research and wrote the paper. YC and KA performed the research. YO contributed the transgenic mice. YC, KA, MKK, and MK analyzed the data.

## Conflict of Interest Statement

The authors declare that the research was conducted in the absence of any commercial or financial relationships that could be construed as a potential conflict of interest.

## Abbreviations

AAV: adeno-associated virus
CP: choroid plexus
CSF: cerebrospinal fluid
LM: leptomeninges
LPGDS: lipocalin-type PGDS
OD: oligodendrocytes
PGD_2_: prostaglandin D_2_
PGDS: PGD synthase
REM: rapid-eye movement
