# Supplementary material for: The Leptomeninges Produce Prostaglandin D2 Involved in Sleep Regulation in Mice
Source: Front Cell Neurosci. 2018 Oct 11;12:357. doi: 10.3389/fncel.2018.00357 (PMC6193105; doi:10.3389/fncel.2018.00357)
Supplement: Supplementary file 1 [file Data_Sheet_1.docx]

Supplementary Material

The leptomeninges produce prostaglandin D_2_ involved in sleep regulation in mice

Yoan Cherasse^*^, Kosuke Aritake, Yo Oishi, Mahesh K. Kaushik, Mustafa Korkutata, Yoshihiro Urade^*^

***Correspondence:**

Prof. Yoshihiro Urade

uradey@gmail.com

Dr. Yoan Cherasse

Cherasse.yoan.fm@u.tsukuba.ac.jp

## Supplementary Figures

**
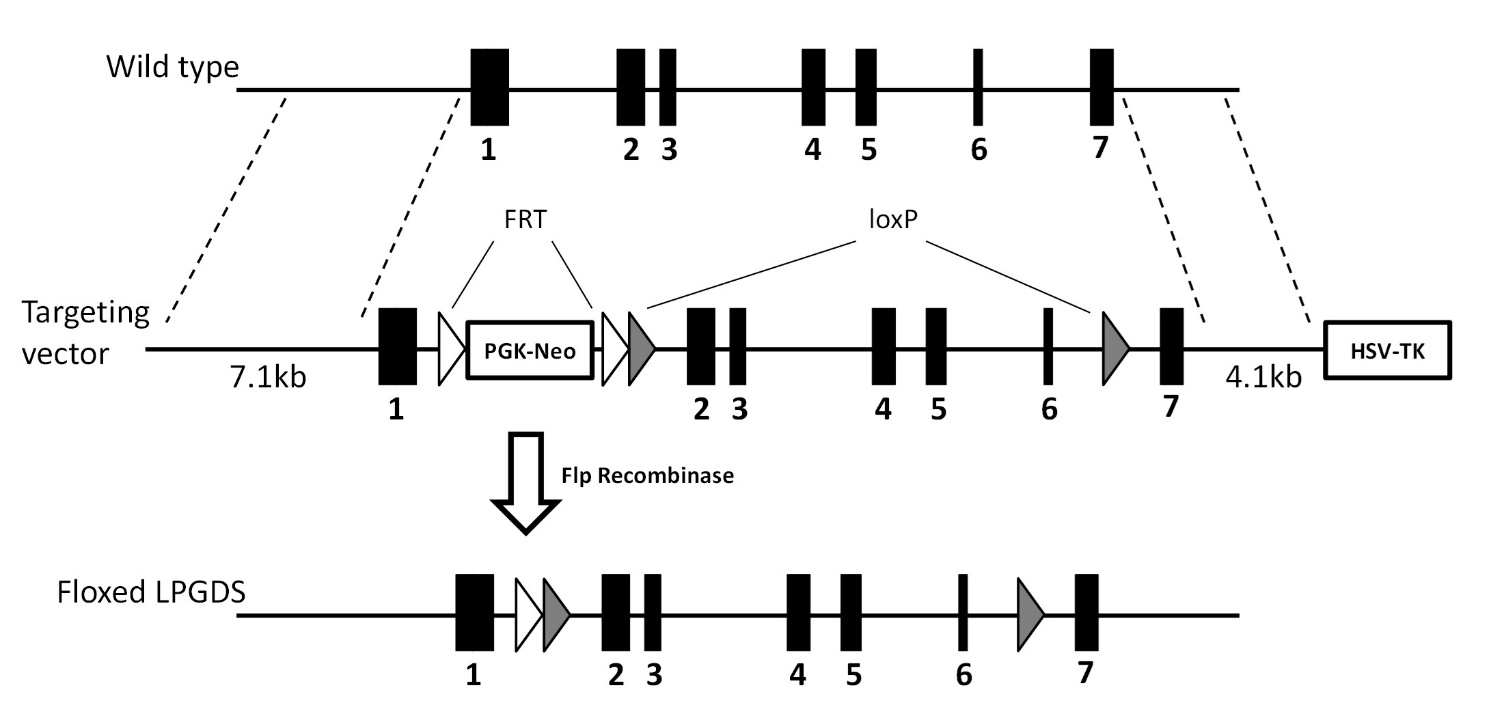
**

**Supplementary Figure 1.** Strategy for the generation of a Flex-LPGDS mouse line.


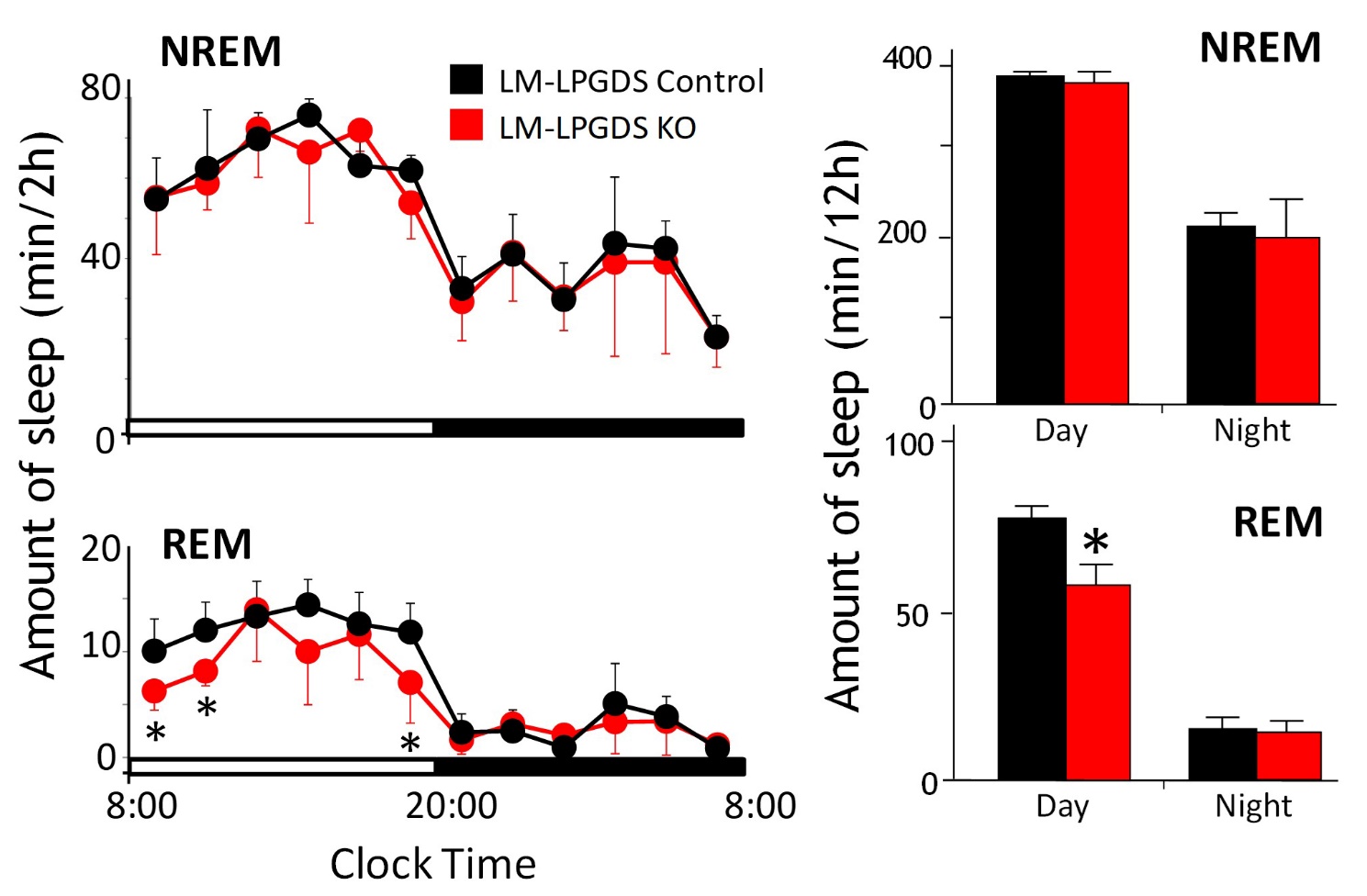


**Supplementary Figure 2.** Baseline sleep activity of LM-LPGDS KO mice. The total amount of NREM sleep is similar between the LM-LPGDS KO mice and the control mice over 24 hours recording, however the amount of REM sleep was significantly reduced during daytime in the KO mouse. *p<0.05, LM-LPGDS KO vs Control, n=6.

**
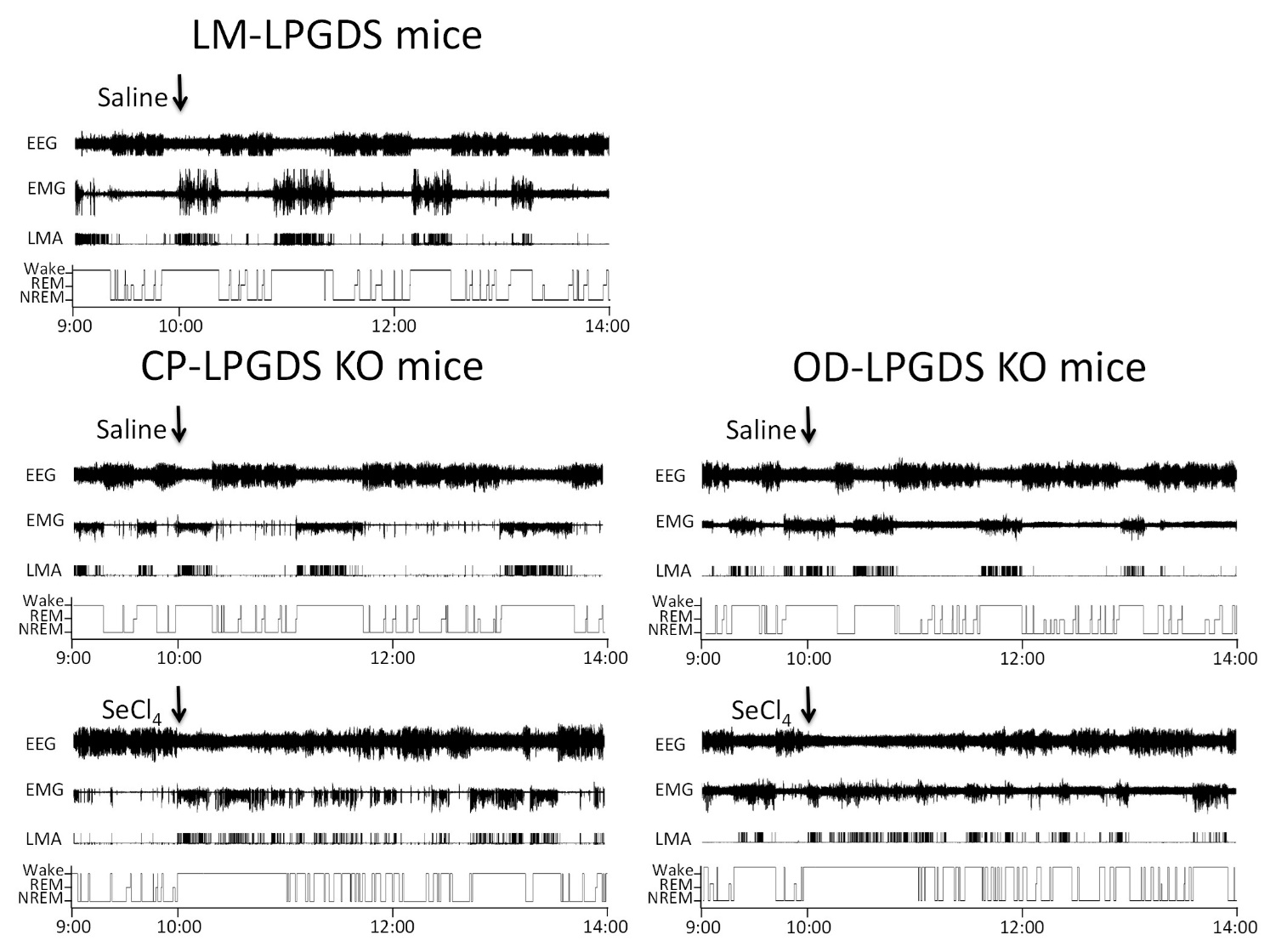
**

**Supplementary Figure 3.** EEG, EMG, locomotor recordings and hypnograms for 4 hours following saline administration in LM- CP and OD-LPGDS KO mice as well as 4 hours following SeCl_4_ administration in CP- and OD-LPGDS KO mice.
